# Molecular docking as a tool for the discovery of molecular targets of nutraceuticals in diseases management

**DOI:** 10.1038/s41598-023-40160-2

**Published:** 2023-08-17

**Authors:** P. C. Agu, C. A. Afiukwa, O. U. Orji, E. M. Ezeh, I. H. Ofoke, C. O. Ogbu, E. I. Ugwuja, P. M. Aja

**Affiliations:** 1 Department of Biochemistry, Faculty of Sciences, Ebonyi State University, Abakaliki, Nigeria; 2 Department of Biotechnology, Faculty of Sciences, Ebonyi State University, Abakaliki, Nigeria; 3 Department of Chemical Engineering, Faculty of Engineering, Caritas University, Amorji-Nike, Enugu, Nigeria; 4 Department of Biochemistry, Faculty of Sciences, Madonna University, Elele, Rivers State Nigeria; 5 Department of Biochemistry, Federal University of Health Sciences, Otukpo, Benue State Nigeria; 6 Department of Science Laboratory Technology (Biochemistry Option), Our Savior Institute of Science, Agriculture, and Technology, Enugu, Nigeria; 7 Department of Biochemistry, Faculty of Biomedical Sciences, Kampala International University, Ishaka, Uganda

**Keywords:** Pharmaceutics, Target identification, Target validation

## Abstract

Molecular docking is a computational technique that predicts the binding affinity of ligands to receptor proteins. Although it has potential uses in nutraceutical research, it has developed into a formidable tool for drug development. Bioactive substances called nutraceuticals are present in food sources and can be used in the management of diseases. Finding their molecular targets can help in the creation of disease-specific new therapies. The purpose of this review was to explore molecular docking's application to the study of dietary supplements and disease management. First, an overview of the fundamentals of molecular docking and the various software tools available for docking was presented. The limitations and difficulties of using molecular docking in nutraceutical research are also covered, including the reliability of scoring functions and the requirement for experimental validation. Additionally, there was a focus on the identification of molecular targets for nutraceuticals in numerous disease models, including those for sickle cell disease, cancer, cardiovascular, gut, reproductive, and neurodegenerative disorders. We further highlighted biochemistry pathways and models from recent studies that have revealed molecular mechanisms to pinpoint new nutraceuticals' effects on disease pathogenesis. It is convincingly true that molecular docking is a useful tool for identifying the molecular targets of nutraceuticals in the management of diseases. It may offer information about how nutraceuticals work and support the creation of new therapeutics. Therefore, molecular docking has a bright future in nutraceutical research and has a lot of potentials to lead to the creation of brand-new medicines for the treatment of disease.

## Introduction

Molecular Docking has become an essential aspect of in-silico drug development in recent years. This technique involves predicting the interaction between a small molecule and a protein at the atomic level^1^. This enables researchers to study the behavior of small molecules, such as nutraceuticals, within the binding site of a target protein and understand the fundamental biochemical process underlying this interaction^2^. The technique is structure-based and requires a high-resolution 3D representation of the target protein obtained through techniques like X-ray crystallography, Nuclear Magnetic Resonance Spectroscopy, or Cryo-Electron Microscopy^3–5^.

There are several computational tools and algorithms available for molecular docking techniques, both commercial and free-of-charge. These programs and tools have been developed and are currently being used in drug research and academic fields^6–9^. According to Sahoo et al.^1^ some of the most commonly used docking programs include AutoDock Vina, Discovery Studio, Surflex, AutoDock GOLD, Glide, MCDock, MOE-Dock, FlexX, DOCK, LeDock, rDock, ICM, Cdcker, LigandFit, FRED, and UCSF Dock. Among these programs, AutoDock Vina, Glide, and AutoDock GOLD have been identified as top-ranking choices with the best scores^10–12^. Additionally, some of these programs have been effective in predicting Root Mean Square Deviations (RMSDs) ranging from 1.5 to 2 Å, depending on the experimental poses^1^. However, flexible receptor docking, specifically receptor backbone flexibility, remains a challenge for contemporary docking programs^13^.

The ligand-receptor complex's computational electrostatics can be assessed, screened, and predicted through the docking study, as stated by Sahoo et al*.*^1^. This study typically follows two distinct steps, according to Mohapatra et al*.*^14^. First, ligand conformations are sampled according to the protein's active site. Second, the conformations are ranked according to a scoring function. Sampling algorithms should theoretically reproduce experimental binding modes, and the confirmations obtained should be ranked according to a scoring function, as per Dash et al*.*^15^. The dry lab approach offers a significant advantage over in vivo lab studies in terms of resource and time investment, as noted by Sahoo et al*.*^16^ and Pramanik et al*.*^17^. Nanda et al*.*^18^ explained that this approach predicts the ligand orientation in a complex formed by the ligand itself with proteins or enzymes. Also, the docked complex's shape and electrostatic interaction quantify the interaction.

The utility of Molecular Docking in drug discovery and design has been well-established^19^. However, Tao et al*.*^20^ have recently reported a surge of interest in the application of this method in food science. Specifically, molecular docking is being utilized to authenticate the molecular targets of nutraceuticals in disease management^21^.

Nutraceuticals are natural substances with therapeutic benefits on human health that are often sourced from dietary sources^22^. Because of their potential to prevent and control chronic diseases including cancer, diabetes, cardiovascular, and neurodegenerative disorders, these have grown in popularity over the past several years^23–25^. Before in vitro investigations, molecular docking studies are used in the field of nutraceutical research to provide crucial information^26^. The goal of this review is to examine the most pertinent molecular docking applications for assessing the possible health-promoting effects of nutraceuticals.

### Molecular docking

#### Molecular docking rudiments

Molecular docking aims to predict the ligand-receptor complex through computer-based methods^27^. The process of docking involves two main steps which include sampling the ligand and utilizing a scoring function^28^. Sampling algorithms help to identify the most energetically favorable conformations of the ligand within the protein's active site, taking into account their binding mode. These confirmations are then ranked using a scoring function^7,28^.

#### Search algorithms

**Figure 1:**
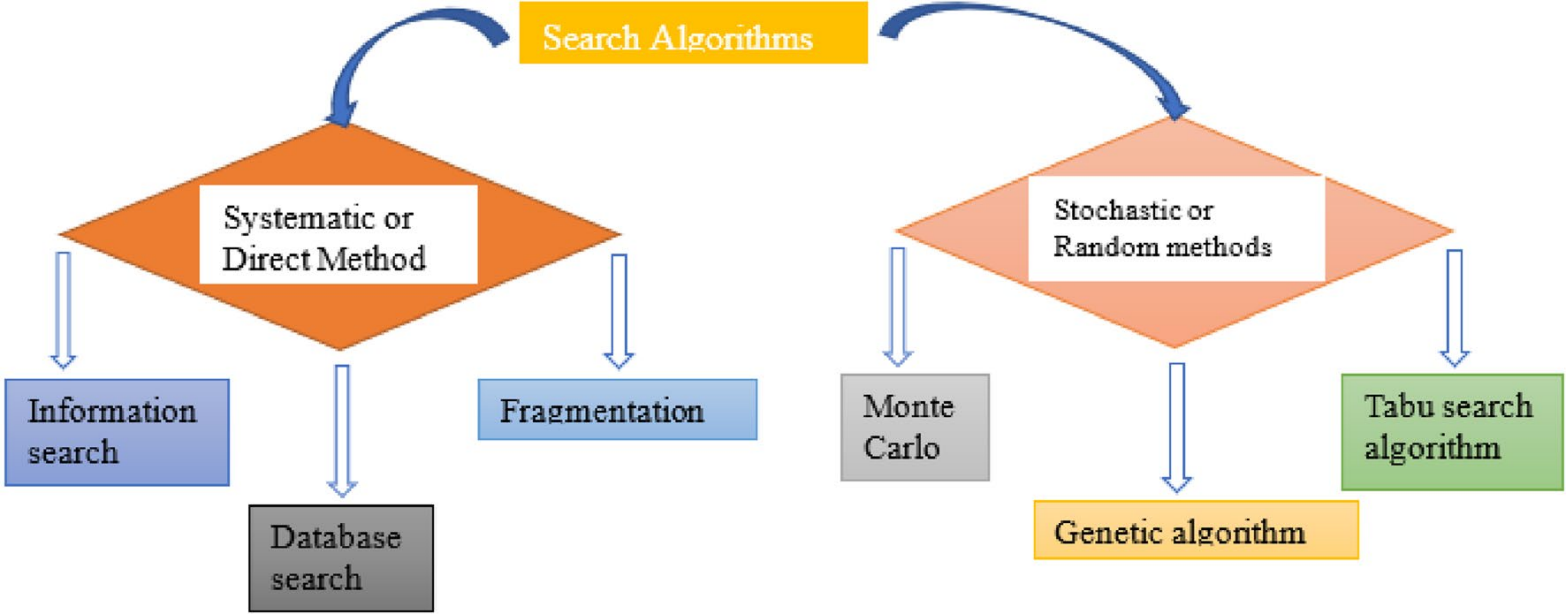
Classes of search algorithm mechanisms.

The principal target of the search algorithm is to locate every single imaginable direction and conformation of the protein combined with the ligand^28^. The search algorithms are classified as shown in Fig. 1.

- *Systematic or direct method* There are three subtypes of systematic methods as follows:
  i. *Conformational search* Here, the torsional (dihedral), translational, and rotational degrees of freedom of the ligand's structural parameter is gradually changed^29,30^.
  ii. *Fragmentation* Here, multiple fragments may be docked during the molecular docking process to form bonds between them, or the fragments may be anchored separately, with the first fragment being docked first and subsequent fragments being built outward in steps from that initial bound position. It uses the tools like Flex XTM, DOCK, LUDI, etc.
  iii. *Database Search* By using this technique, it is possible to create many reasonable conformations of every tiny molecule that is already recorded in the database and then dock them as hard bodies. FLOG is an example of the tools it uses.
- *Stochastic methods or Random methods* There are three subtypes of stochastic methods.
  i. *Monte Carlo* This approach involves arbitrarily placing ligands in the receptor binding site, scoring it, and then generating a new configuration. It employs instruments such as MCDOCK, ICM, etc.
  ii. *Genetic algorithm* It starts with a population of postures, where the configuration and location concerning the receptor are described by the “gene” and the score is the "fitness." Perform transformations, hybrids, etc., of the fittest to produce the next generation and repeat the agreement^31,32^. It uses programs like GOLD, AutoDock, and others.
- *Tabu search* It operates by striking limitations that facilitate the research of a fresh configuration by preventing the previously exposed areas of the ligands’ conformational space from being examined again. The tools it uses are PRO LEADS, Molegro Virtual Docker (MVD)TM, etc.

#### Scoring functions

**Figure 2:**
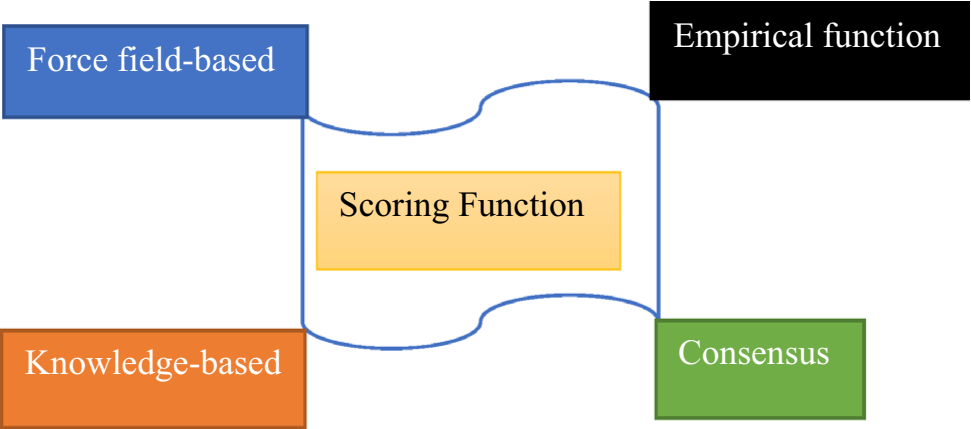
Classes of scoring function mechanisms.

By applying virtual screening, ligands are evaluated according to their binding affinity, which aids in the evaluation of which ligand structure and rotation is most advantageous concerning the receptor (protein)^28,31^. According to Fig. 2, four main groupings make up the scoring function.

- *Force field-based* In an ace capacity, it adds the contribution of non-bonded interactions including van der Waal forces, hydrogen bonding, and Columbic electrostatics as well as bond-like angle bonding and torsional deviation to determine the binding affinity^33^. Tools like AutoDock, DOCK, GoldScore, etc. are employed.
- *Empirical function* It relies on repeated linear relapse analysis of a prepared set of complex structures using protein–ligand complexes with known binding affinities, comprising functional groups and some type of interaction. Examples include the N–O hydrogen link, the O–O hydrogen bond, the salt scaffold, the stacking of aromatic rings, etc.^34^. It makes use of technologies like LUDI score, ChemScore, AutoDock scoring, etc.
- *Knowledge-based* By statistically assessing a collection of complex structures, it provides elements, atoms, and functional groupings with the possibility for separation into ward pairs^35^. Instruments like PMF and DrugScore are employed.
- *Consensus* Fundamentally, it fuses the evaluations or orders acquired through multiple evaluation methods in various arrangements^28^.

### Molecular docking software

#### Molecular docking program design

In many drug discovery initiatives, molecular docking has become crucial, especially for the virtual screening of phytochemicals or nutraceuticals as possible therapeutic compounds^7^. Irwin Kuntz of the University of California created the first docking program in the middle of the 1980s, and efforts are constantly being made to enhance docking computations. Current developments in docking techniques identify an enzyme’s natural substrates to forecast its capacity^36^. By determining that the protein of interest belongs to a certain superfamily, protein complexes can be successfully predicted by limiting the search for probable substrates and reaction types to that region^37^.

#### Methodologies of ranking docked molecules

The docked molecules are carefully ranked using a variety of approaches and systems. This section highlights the often used.

##### DOCK 3.5.x

The idea behind this program is that enzymes catalyze processes by restricting the transition state that is preferable to the substrate. Furthermore, amidohydrolase superfamily hydrolysis processes the protein to maintain its stiffness, therefore docking molecules that match transition states should yield a stronger signal than docking substrates^38^.

##### Glide

The program distinguishes the enzymes having a place with a specific subgroup of the enolase superfamily allowing tapering the arrangement of potential substrates, and precision in the positioning was improved by fine-tuning and rescoring the docked complex with an increasingly perplexed material science-based scoring capacity and allowing receptor side chains to move^39^.

#### Highlights of molecular docking software

Many programs are available for docking, and some of the most popular ones are discussed in this section.

##### Dock

Dock is a molecular docking software developed by the UCSF Chimera team. It is a user-friendly tool that can be used to dock small molecules into a receptor-binding site. Dock uses a grid-based method to evaluate the binding affinity of ligands to the receptor. It also includes scoring functions to rank the poses generated during the docking process. The dock supports several input file formats, including PDB, MOL2, and SDF. The dock is available from http://dock.compbio.ucsf.edu/.

##### Autodock

Autodock is a widely used molecular docking software developed by the Scripps Research Institute. It is a free, open-source software that can perform both rigid and flexible docking. Autodock uses a Lamarckian genetic algorithm to optimize the placement of ligands within a receptor binding site. It also includes several scoring functions to evaluate the binding affinity of ligands to the receptor. Autodock supports a variety of input file formats, including PDB, MOL2, and SDF. AutoDock is available from http://autodock.scripps.edu

##### Argus lab 4.0.1

Argus lab, a molecular modeling software created by Mark Thomson of the Department of Energy at Pacific Northwest National Laboratory in the USA, utilizes a combination of quantum mechanics and classical mechanics algorithms to model solvent effects. This software is capable of performing tasks such as drug design, producing graphics, and molecular modeling. Argus lab is available at http://www.arguslab.com.

##### Genetic optimization for ligand docking (GOLDTM)

GOLD is a protein–ligand docking software that offers several key features. It allows for the inclusion of spine and side chain adaptability in computations and uses user-defined scoring functions that can adapt accordingly. The energy functions are based on both conformational and non-reinforced contact information. There are also various options available for docking, including the ability to remove crystallographic water molecules in the ligand binding site. Additionally, GOLD can handle metal atoms automatically if they are correctly set up in the protein data file. Finally, virtual screening high-throughput screening results can be analyzed and post-processed efficiently using the companion programs SILVERTM or GoldMineTM. The latest version of GOLD Suite 5.2 includes three components: Gold 5.2 for protein–ligand docking, Hermes 1.6 for comprehensive protein visualization, and Gold Mine 1.5 for analysis of docking grades. This software is available at http://www.ccdc.cam.ac.uk/ products/lifesciences/gold.

##### MolDock

MolDock is a molecular docking software developed by MolSoft LLC^40^. It is a fast and efficient docking program that can be used to dock small molecules into a receptor-binding site. MolDock uses a fast Fourier transform (FFT) algorithm to evaluate the binding affinity of ligands to the receptor. It also includes a scoring function that takes into account the shape complementarity, electrostatic interactions, and van der Waals forces between the ligand and the receptor. MolDock supports several input file formats, including PDB, MOL2, and SDF. It is available at https://www.molsoft.com/about.html.

##### Discovery studio

Discovery Studio is a software package for molecular modeling and simulation, developed by Dassault Systèmes BIOVIA. It includes a range of tools for molecular docking, virtual screening, protein modeling, and analysis of molecular dynamics simulations. The molecular docking component is used to predict the binding mode of a ligand (small molecule) to a target protein and to estimate the strength of the interaction between them. Discovery Studio employs a variety of docking algorithms, such as CDOCKER, GOLD, and LibDock, to generate a set of possible binding poses for the ligand, and to rank them based on their predicted binding energy. The software also provides tools for visualizing and analyzing the docking results, and for comparing the binding modes of different ligands to the same protein target. It is available at https://discover.3ds.com/discovery-studio-visualizer-download.

##### Chimera

Chimera is a software package for visualizing, analyzing, and modeling molecular structures, developed by the University of California, San Francisco. It provides a range of tools for displaying 3D structures of proteins, nucleic acids, and small molecules, and for performing molecular docking simulations. The molecular docking component in Chimera is called “Dock Prep” and is used to prepare the ligand and target protein for docking simulations. It includes tools for adding hydrogens, assigning charges, and generating molecular surfaces, which can be used to guide the placement of the ligand in the binding site of the protein. Chimera also provides tools for analyzing the docking results, such as visualizing the binding poses, calculating binding energies, and generating interaction maps between the ligand and protein residues. Additionally, Chimera can interface with other molecular docking software packages, such as AutoDock, to perform more advanced docking simulations. Chimera is available from https://www.cgl.ucsf.edu/chimera/.2.4

### Representation of molecular docking

**Figure 3:**
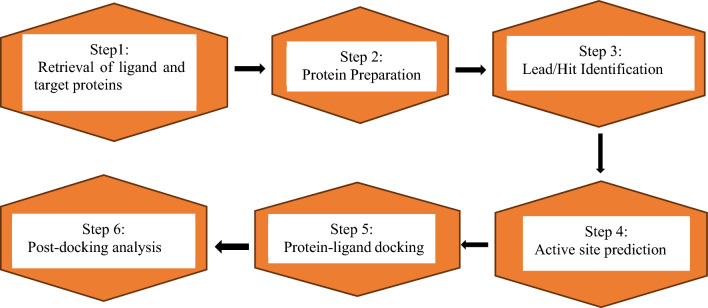
A prototype flow chart of a molecular docking study.

Generally, the Docking process can be represented in a flowchart as shown in Fig. 3.

#### Retrieval of protein and ligand structure

Finding practical target proteins and ligands is crucial for carrying out docking. Thus, one must check to see if the target protein has been stored in the Swiss UniProt knowledge base (http://expasy.org/sprot) or the Protein Data Bank (PDB) database (http://pdb.org). Homology modeling may be done utilizing the Swiss model repository (http://swissmodel.expasy.org/repository/), modeller programs like I-TASSER, etc. if the target protein is not in the database but comparable sequences are. The next step is to locate the ligand using the PubChem database (http://pubchem.org), Zinc (http://blaster.docking.org/zinc/), ChmBl, or if none of these databases include the ligand, ChemDraw or ChemSketch can be used to synthesize the ligand from scratch^41^.

#### Protein preparation

The process of optimizing the protein structure and getting it ready for precise docking simulations is known as protein preparation, and it is an essential stage in the molecular docking process. The protein structure is first obtained from a database like Protein Databank (PDB) or created using molecular modeling tools like SWISS MODELLER. The structure is then completed by adding any extra atoms or residues. The protein is subsequently put through energy minimization to loosen up the structure and get rid of any steric interference. The protonation states of ionizable residues are then established to provide proper electrostatic interactions during docking. To further simplify the system, water molecules, and extraneous ligands are eliminated from the protein structure. To appropriately reflect the protein's behavior during docking simulations, the proper force field parameters are lastly supplied to it. Through these preparation steps, the protein structure is optimized and refined, providing a suitable starting point for successful molecular docking studies^42^.

#### Lead or hit identification

The ligands to be docked are selected based on various criteria, such as their chemical diversity, their known biological activity, or their potential for drug development. The ligands are then prepared for docking by assigning charges, generating conformers, and optimizing their geometry^40,42^.

#### Active site prediction

During molecular docking, the binding pockets can be initially specified or identified after docking. Consequently, to validate the binding pocket of interest during molecular docking three different approaches can be conceived^28^ as highlighted below.

i. *Site-directed docking* Here, first, identify the protein–ligand binding site and then dock the ligand.
ii. *Blind docking* Here, the docked ligand is directly onto the complete receptor structure without prior knowledge of the binding site^43^.
iii. *Docking with a standard* Here, you dock the protein with the test ligands and/or standard small molecule(s)^41^. The standard ligand facilitates the prediction of the relevant binding pocket.

Additionally, calculating the inhibition constant for docked ligands and proteins is an important step in evaluating the binding affinity and potential inhibitory activity of the ligands. However, it is not always necessary or applicable in all cases. The decision to calculate the inhibition constant depends on the specific research question, experimental design, and the objectives of the study.

#### Protein–ligand docking

Ligand is docked against the protein and the interactions are analyzed. The scoring function gives a score based on the best-docked ligand complex picked out^44^.

#### Post-docking analysis

After the ligands have been docked to the protein, the results are analyzed to identify the most promising candidates for further study. The binding affinity of each ligand is calculated based on the predicted interaction energy, and the ligands are ranked based on their affinity scores. The docked structures are also analyzed to identify key interactions between the ligands and the protein, such as hydrogen bonds, hydrophobic interactions, and electrostatic interactions. These interactions can provide insights into the mechanism of action of the ligands and guide further optimization of their structure^19^.

### General application of molecular docking

#### Hit identification/virtual screening

Molecular docking is widely used in hit identification in drug discovery. It helps in identifying potential drug candidates by predicting the binding affinity of small molecules to a protein or receptor of interest. Docking can be used to screen a large database of small molecules to identify those that can bind to a protein of interest with high affinity^45^.

#### Lead optimization

Once a hit compound is identified, molecular docking can be used to optimize the lead compound's structure to improve its binding affinity and selectivity. Docking can also be used to design new analogs by predicting the binding modes of modified structures^46^.

#### Bioremediation

Molecular docking is used in bioremediation to predict the binding affinity of small molecules to enzymes involved in the degradation of environmental pollutants. Docking can help in designing inhibitors or activators of these enzymes to enhance bioremediation efficiency^47^.

#### ADMET prediction

Docking can also be used to predict the Absorption, Distribution, Metabolism, Excretion, and Toxicity (ADMET) properties of small molecules. The predicted ADMET properties can be used to screen out compounds with unfavorable properties early in the drug discovery process^28^. Some notable examples include AutoDock Vina, GOLD (Genetic Optimization for Ligand Docking), Glide, and Schrödinger Suite. These software packages provide advanced algorithms and computational techniques for efficient ligand-receptor docking simulations, allowing for the prediction of binding affinities and identifying potential drug candidates. Furthermore, they incorporate ADMET prediction modules, enabling the assessment of the drug's behavior in terms of its absorption, distribution within the body, metabolism, excretion, and potential toxicity.

#### Molecular dynamics simulation

Molecular docking can be combined with molecular dynamic simulations to study the dynamic behavior of protein–ligand complexes. The simulations can help in understanding the conformational changes that occur upon ligand binding and the stability of the complex^48^. Several software tools combine molecular docking and dynamics simulation. These include frequently used software like AutoDock, Vina, Glide, and GOLD. In addition to molecular docking, they provide capabilities for conducting molecular dynamics simulations, allowing for the exploration of protein–ligand interactions over time and the analysis of their dynamic behavior.

#### Structure elucidation

Molecular docking can also be used to elucidate the structure of proteins with unknown structures. Docking can be used to predict the binding modes of small molecules to the protein and generate a homology model of the protein based on the binding mode prediction. The generated model can then be refined using experimental data to obtain an accurate structure of the protein^49^.

## Nutraceuticals

### Concept of nutraceuticals

In 1989, a term called “nutraceutical” was coined by DeFelice and the Foundation for Innovation in Medicine, combining the words “nutrition” and “pharmaceutical”^50^. A press release in 1994 defined nutraceuticals as substances that provide medical or health benefits and can be considered food or a component of food, including disease prevention and treatment^51,52^. As further explained by Raj et al*.*^53^, nutraceuticals can include a wide range of substances, such as isolated nutrients, dietary supplements, diets, herbal products, genetically engineered designer foods, and processed foods like cereals, soups, and beverages.

Despite their distinctions, nutraceuticals and functional foods are occasionally used interchangeably. Nutraceuticals as well as functional foods are two phrases used to refer to food items that offer extra health advantages over and beyond basic nutrition^52,54^. Nutraceuticals are isolated chemicals that have been taken from food sources and are sold in supplement form^50,55^, whereas functional foods are entire foods that have been fortified or augmented with nutrients or bioactive components that give a specific health benefit^24,56^. Nutraceuticals are frequently categorized as dietary supplements and are subject to different laws than functional foods in many nations^50,56^.

The idea of nutraceuticals is fundamentally influenced by personal interests and degree of expertise. Cardiologists could, for instance, prioritize dietary supplements that have been linked to decreasing heart diseases risk factors, such as those that have a positive effect on hypertension, hypercholesterolemia, and the decrease of free radical or platelet-dependent thrombotic activity^57^. Phytosterols, N-3 fatty acids, quercetin, and grape flavonoids are of particular significance to cardiologists^24^. On the other hand, oncologists may focus on nutraceuticals that have anticarcinogenic effects, such as those that enhance antioxidant and microsomal detoxification systems or decrease the growth of cancer that has already manifested^56^.

### Classifications of nutraceuticals

**Figure 4:**
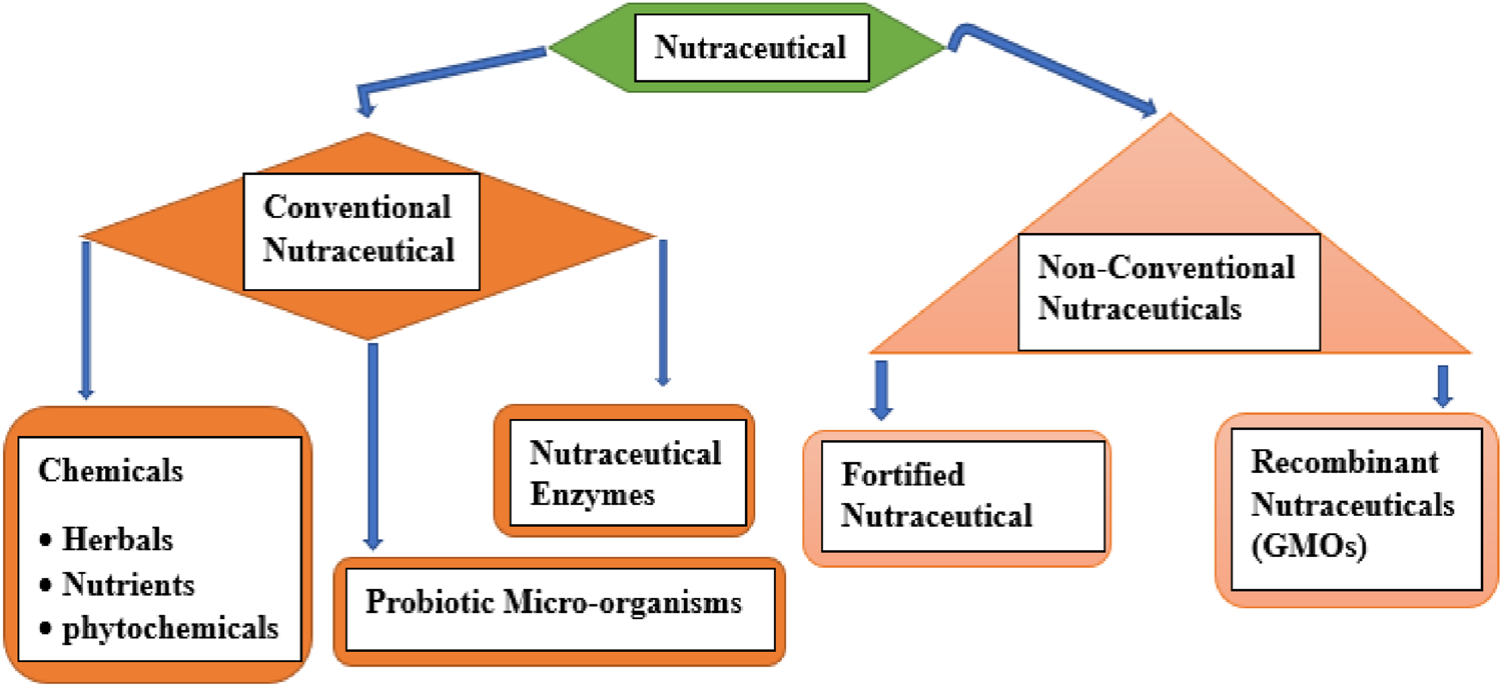
Classification of nutraceuticals^22^.

Several authors used different methods to classify nutraceuticals. However, this seminar considers the food availability framework according to Bairagi and Patel^22^. Therefore, nutraceuticals are classified as shown in Fig. 4.

#### Conventional nutraceuticals

Nutraceuticals that have been extensively researched for their health benefits and are widely recognized as traditional include vitamins, minerals, herbal extracts, and plant-based supplements^58^. These conventional nutraceuticals have been in use for centuries for their medicinal properties and are readily available in the market^59^. Conventional nutraceuticals such as vitamin C, vitamin D, omega-3 fatty acids, and probiotics have been widely accepted^22^.

- *Chemicals (Nutrients, Herbals, and Phytocompounds)* Nutraceuticals encompass a variety of compounds, with chemicals being one of the most commonly studied^22^. These chemicals can be further classified into three distinct groups: nutrients, herbals, and phytocompounds. Nutrients, such as vitamins and minerals, play a crucial role in maintaining normal bodily functions^60^. Herbals are derived from plant sources and are thought to provide various health benefits. Examples of herbals include ginger, turmeric, and ginseng. Phytocompounds, on the other hand, refer to bioactive compounds found in plants that are believed to have therapeutic properties. Prominent examples of phytocompounds include polyphenols, flavonoids, and carotenoids^61^.
- *Probiotic micro-organisms* Live bacteria known as probiotics are said to have health advantages when ingested. Typically, they may be discovered in fermented foods like yogurt, kefir, and sauerkraut. Probiotics are supposed to promote gut health by balancing out the beneficial bacteria present in the gut microbiome. Moreover, they could give the immune system the vitality required to reduce inflammation^22^.
- *Nutraceutical enzymes* Proteins called enzymes speed up chemical processes in the body. Enzymes that are considered to offer therapeutic advantages when taken as supplements are known as nutraceutical enzymes. For instance, proteolytic enzymes may assist lower inflammation in the body while digestive enzymes may aid enhance food digestion^56^.

#### Non-conventional nutraceuticals

Unconventional sources such as algae, fungi, and animal by-products have given rise to a new generation of less familiar nutraceuticals^22,56^. Among these non-conventional nutraceuticals are exotic fruits, novel proteins, and unique bioactive compounds. Despite the growing interest in these nutraceuticals, their potential health benefits, safety, and efficacy are still under investigation^61^. Spirulina, chlorella, mushroom extracts, and insect-based proteins are examples of non-conventional nutraceuticals that have emerged in recent years^22,24,59^.

- *Fortified Nutraceuticals* To enhance the nutritional value of products, fortified nutraceuticals are enriched with extra vitamins, minerals, and nutrients. This process is aimed at providing more health benefits beyond their natural form. As stated by Rajasekaran and Kalaivani^62^, “fortified nutraceuticals are defined as food or food products that are fortified with additional nutrients to provide a health benefit beyond their normal nutritional content”. Some examples of fortified nutraceuticals include:
  i. *Fortified fruit juices* These drinks include added vitamins and minerals to improve their nutritional value. For instance, calcium and vitamin D may be added to orange juice to promote the health of your bones.
  ii. *Fortified breakfast cereals* These are fortified cereals containing added vitamins and minerals. For instance, some cereals could be iron-fortified to guard against iron deficiency anemia.
  iii. *Fortified milk* Milk may be fortified with vitamin D to support bone health and calcium absorption.
  iv. *Fortified energy drinks* To boost energy metabolism, energy drinks may be supplemented with vitamins and minerals.
- *Recombinant Nutraceuticals* Innovative nutraceuticals are novel products obtained from genetically engineered organisms (GMOs) that are programmed to synthesize targeted nutrients or bioactive molecules. These novel products are intended to confer extra health benefits beyond the native versions^63^. A few instances of innovative nutraceuticals comprise:
  i. *Recombinant antibodies* These are antibodies that are produced using recombinant DNA technology. For example, recombinant monoclonal antibodies are used to treat cancer and autoimmune diseases.
  ii. *Recombinant vitamins* These vitamins are created by the use of recombinant DNA technology. Recombinant vitamin B12, for instance, is used to treat vitamin B12 insufficiency.
  iii. *Recombinant proteins* Recombinant DNA technology is employed to produce proteins such as human insulin, which is utilized in the management of diabetes^22^.
  iv. *Recombinant enzymes* Recombinant DNA technology is employed to create enzymes like lactase, which are used to break down lactose in individuals with lactose intolerance^64^.

### Industrial dynamics of nutraceuticals

#### Cannabis blueprint

The popularity of cannabis together with its byproducts in nutraceuticals is on the rise, as they are believed to offer a wide range of health benefits^65^. In a surprising move, the Cannabis Act of 2018 was passed, allowing for the legal cultivation of hemp and marijuana and their derivative products in the United States^22^. Nutraceuticals containing cannabidiol, the active ingredient derived from cannabis or hemp, are becoming increasingly popular^66^. As cannabis becomes legal in more countries, further research is being conducted to determine the potential benefits and risks of cannabis-based products. It is expected that the trend toward cannabis-based nutraceuticals will continue to grow as more consumers become aware of their potential health benefits^64,66^.

#### Nutricosmetics

According to Bairagi and Patel^22^, nutricosmetics are supplements aimed at enhancing the health and appearance of the hair, skin, and nails. As consumers increasingly prioritize natural and holistic approaches to augment their physical appearance, the demand for nutricosmetics is expected to surge. Taeymans et al*.*^67^ suggest that this trend will continue to gain momentum as consumers become more health-conscious and seek multifaceted benefits from the products they purchase.

#### Problem of plastic packaging

The nutraceutical industry is currently grappling with the challenges arising from plastic packaging, which has emerged as a significant worry for both producers and consumers^22,53^. With increasing awareness about the environment, more consumers are seeking out eco-friendly alternatives for packaging^68^. Consequently, manufacturers are devising innovative, sustainable packaging options to cater to this demand^69^.

#### Sports nutrition space

As interest in fitness and sports-related activities increases, so too does the demand for products that can improve athletic performance and overall health. Accordingly, Bairagi and Patel^22^ noted that this trend has led to rapid growth in the sports nutrition market, with no signs of slowing down.

#### Domestic anima food development

Pet owners are increasingly looking for high-quality, nutrient-dense food options for their pets. The trend toward pet food optimization is expected to continue as more consumers become aware of the importance of proper nutrition for their pets^70^.

#### Online marketing of nutraceuticals

The rise of e-commerce and online marketing has made it easier for nutraceutical companies to reach consumers directly. Online marketing allows companies to target specific demographics and provide more personalized marketing messages. This trend is expected to continue as more consumers turn to online shopping for their nutraceutical needs^22^.

#### Seed oil as nutraceutical deposit

Seed oils, such as flaxseed oil as well as chia seed oil are becoming popular as a source of essential fatty acids and other nutrients^71,72^. These oils are being incorporated into a wide range of nutraceutical products, including supplements and functional foods. The trend toward using seed oils as a nutraceutical deposit is expected to continue as consumers seek out more plant-based sources of nutrients^73^.

#### Nutraceuticals against bisphenol A and other endocrine disruptors

Nutraceuticals are gaining popularity as a means of combating the effects of endocrine disruptors such as Bisphenol A (BPA)^74^. BPA is commonly found in plastics, and exposure to it has been linked to a variety of negative health effects, including disruption of the endocrine system^75^. Nutraceuticals such as resveratrol, curcumin, and green tea extract have been shown to have protective effects against BPA-induced damage, with research indicating that they can mitigate the effects of BPA on the body^76,77^. As consumers become more aware of the potential dangers of endocrine disruptors, nutraceuticals are likely to become an increasingly popular choice for those looking to protect their health^77,78^.

Generally, the nutraceuticals industry is experiencing significant growth as consumers become more health-conscious and seek out natural alternatives to traditional pharmaceuticals. Key trends in this industry include the use of innovative delivery methods, the development of personalized nutrition, and the incorporation of technology such as artificial intelligence and blockchain to ensure product quality and traceability. In addition to a rise in interest in the use of nutraceuticals for mental health and wellbeing, there is a rising demand for components that are derived from plants and are sustainably harvested. As the industry continues to evolve, companies that prioritize innovation and sustainability are likely to see the greatest success.

## Molecular docking validations of nutraceuticals targets in diseases

### Theory of molecular docking validation of nutraceuticals

Molecular docking validation is a computational approach that is increasingly being used in the field of nutraceutical research to identify potential targets for the management of various diseases. Nutraceuticals are naturally occurring compounds that have potential health benefits and are found in food sources such as fruits, vegetables, and herbs^73^. With the rise of chronic diseases such as diabetes, cardiovascular disease, and cancer, there is a growing interest in the use of nutraceuticals as a complementary approach to conventional medical treatments^15,22^. Molecular docking validation can help researchers identify potential nutraceutical targets for disease management, providing a more efficient and cost-effective way to screen potential treatments before proceeding with costly clinical trials^79^. This method makes drug discovery more ethical since it lessens the need for animal testing throughout the development of novel medicines^80,81^.

### Molecular docking discovery of nutraceuticals targets

Molecular docking is a computational technique used to predict the interactions between small molecules, such as nutraceuticals, and larger biomolecules, such as enzymes, receptors, RNA, DNA, and other proteins^82^. The technique involves the simulation of the molecular interactions between the small molecule and the target biomolecule, which can provide insights into the binding affinity, binding site, and possible mechanism of action^83^.

#### Enzymes

Enzymes are proteins that catalyze chemical reactions in the body^84^. Nutraceuticals such as curcumin, resveratrol, quercetin, hesperidin, etc., have been shown to interact with enzymes and modulate their activity^21,85^. For example, curcumin has been shown to inhibit the activity of the enzyme COX-2, which is involved in the inflammatory response^86^. Molecular docking can predict the binding site and the strength of the interaction between the nutraceutical and the enzyme, which can help in understanding the mechanism of action and the potential therapeutic benefits^82^.

#### Receptors

Receptors are proteins that bind to specific molecules, such as hormones, neurotransmitters, and drugs, and initiate a cellular response. Nutraceuticals can also interact with receptors and modulate their activity^73^. For example, resveratrol was able to activate the sirtuin family of proteins, which are involved in cellular metabolism and aging^85^. Molecular docking can predict the binding site and the strength of the interaction between the nutraceutical and the receptor, which can help in understanding the mechanism of action and the potential therapeutic benefits^80^.

#### RNA and DNA

Nucleic acids such as RNA and DNA are essential for the storage and transfer of genetic information. By attaching to their structures or changing their expression, nutraceuticals can also interact with RNA or DNA and affect their function^87–89^. For instance, it has been demonstrated that the enzyme topoisomerase, which is involved in DNA replication and repair, is inhibited by quercetin^90^. According to Singh et al*.*^86^, molecular docking can forecast how nutraceuticals may interact with DNA and RNA sequences like telomerase reverse transcriptase (TERT) and microRNAs (miRNAs). Furthermore, molecular docking can forecast the binding location and intensity of the interaction between the nutraceutical and RNA or DNA, which aids in understanding the mechanism of action^91^.

#### Epigenetic markers

According to recent studies^82,92^, epigenetic changes, which can change gene expression without affecting the underlying DNA sequence, are significant targets for nutraceuticals. Molecular docking can predict how nutraceuticals may interact with proteins involved in epigenetic regulation, such as histone deacetylases (HDACs), DNA methyltransferases (DNMTs), and transcription factors/nuclear receptors like estrogen receptors, androgen receptors, fibroblast growth factors^21,93^. These epigenetic modifications can lead to transgenerational effects.

#### Other proteins

Nutraceuticals may potentially target other proteins in the body, including transporters, ion channels, and structural proteins^94^. Hesperidin, for instance, has been demonstrated to block the activity of alpha-glucosidase, an enzyme involved in the breakdown of carbohydrates^21,73^. To understand the mechanism of action and possible therapeutic effects, molecular docking can predict the binding site and the degree of interaction between the nutraceutical and the protein target^91^.

### Applications of nutraceuticals in disease management

#### Nutraceutical in cancer

**Figure 5:**
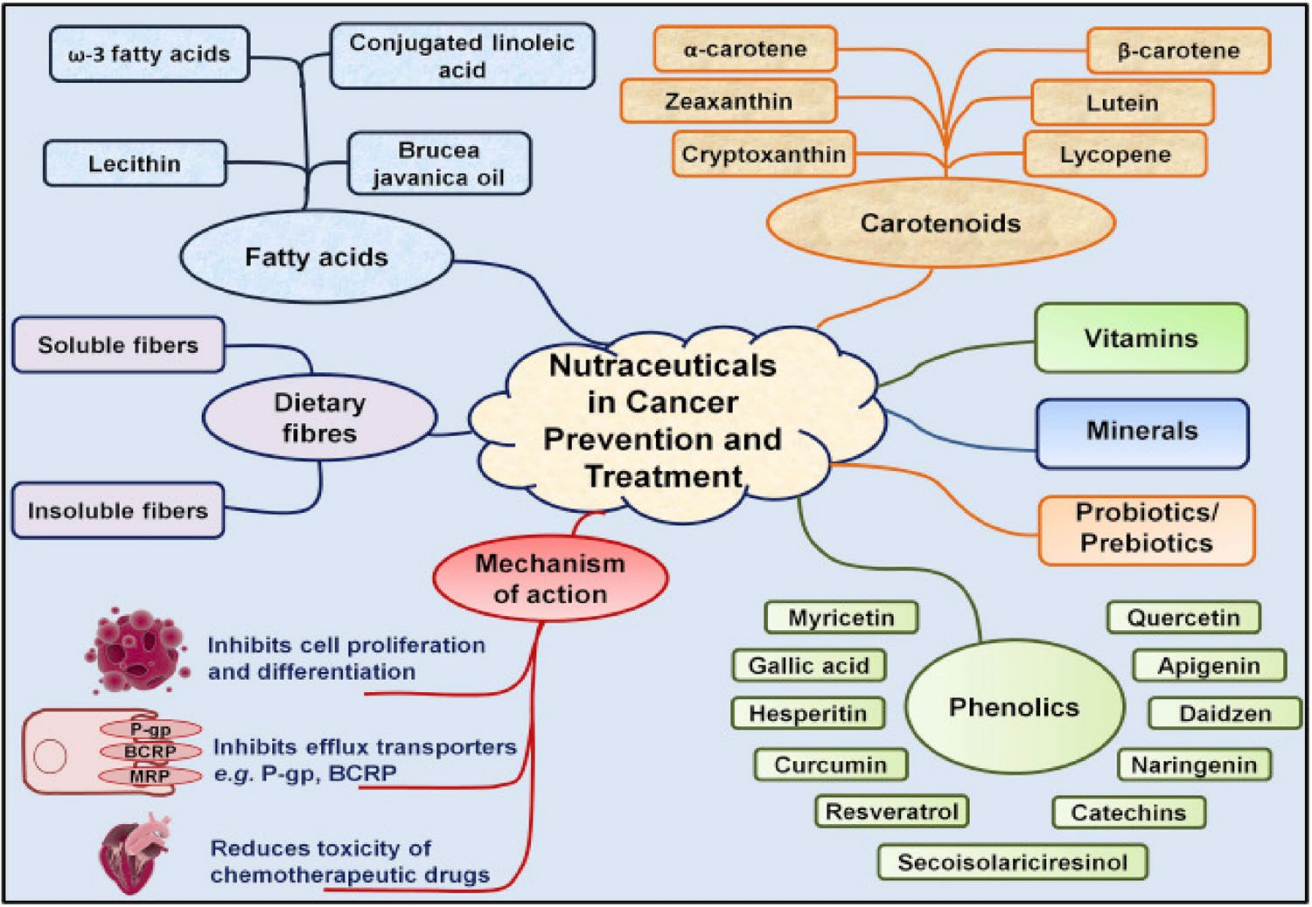
The mechanism of nutraceuticals in cancer management^96^.

Numerous research^95–98^ have demonstrated that some nutraceuticals may have a positive impact on cancer prevention and therapy by regulating important signaling pathways involved in tumor development and metastasis (Fig. 5).

Phytocompounds such as phenolics, flavonoids, and others possess anticancer potentials^41^. One of the most well-known nutraceuticals in cancer prevention is curcumin, a compound found in turmeric^96,98^. As shown in Fig. 5, Curcumin has been shown to inhibit the activation of NF-κB, a transcription factor that plays a critical role in inflammation and cancer development. NF-κB activation can result in the expression of genes that promote tumor growth, invasion, and metastasis^97,99^. By inhibiting NF-κB, curcumin can suppress the development and progression of cancer^95,100^.

Another nutraceutical that has been studied extensively in cancer prevention is resveratrol, a polyphenol found in grapes, berries, and peanuts. The PI3K/Akt/mTOR pathway, which controls cell proliferation and survival, is one of the signaling pathways that resveratrol modulates and is associated with the development of cancer^101^. Resveratrol inhibits the activation of Akt, a kinase that is frequently overexpressed in cancer and can induce cell cycle arrest and apoptosis in cancer cells^97^.

According to research by Melzer et al*.*^102^, green tea polyphenol epigallocatechin-3-gallate (EGCG) may have anticancer properties. The Wnt/-catenin pathway, which is crucial for cell division and proliferation, is one of the signaling pathways that can be inhibited by EGCG^103^. EGCG can also prevent the activation of other signaling pathways that are implicated in the growth of cancer^102^. In addition, EGCG has been shown in animal models to stop tumor development and spread by causing cell cycle arrest and death in cancer cells^100,103^.

Numerous additional substances contained in foods or supplements, in addition to these nutraceuticals, have also been proven to have potential anticancer benefits^104^. These include sulforaphane, a substance found in cruciferous vegetables, which can activate Nrf2, a transcription factor that controls antioxidant and detoxification pathways and can induce apoptosis in cancer cells^96^.

Overall, the use of nutraceuticals in cancer prevention and treatment is an exciting area of research that holds promise for developing novel therapies for this devastating disease. By targeting key signaling pathways involved in tumor growth and metastasis, nutraceuticals have the potential to complement conventional cancer treatments and improve patient outcomes^100^.

#### Nutraceuticals in cardiovascular health

**Figure 6:**
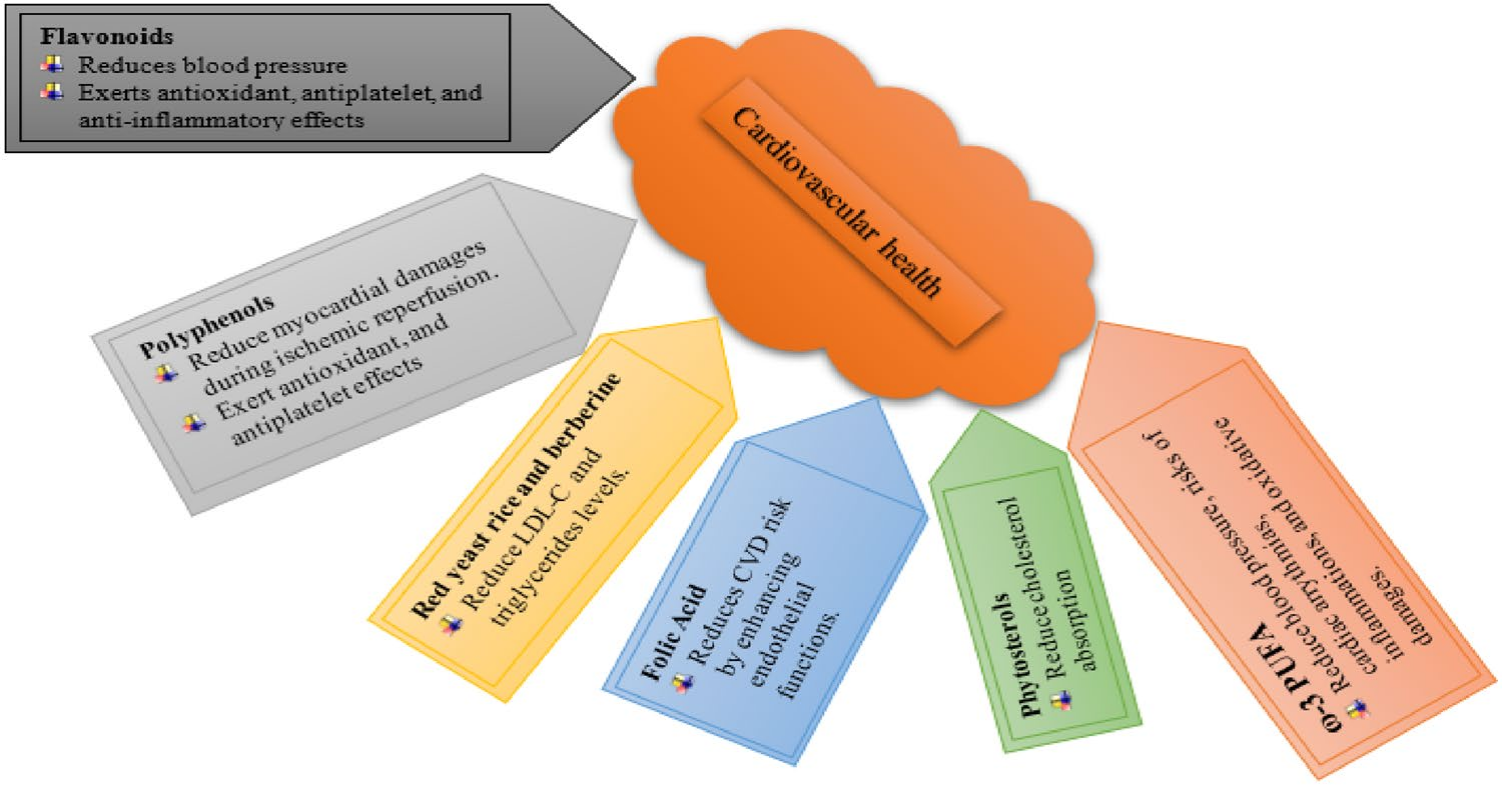
Nutraceutical’s effects on the cardiovascular system.

It has been demonstrated that several nutraceuticals help to promote cardiovascular health by lowering blood pressure, cholesterol levels, and inflammation^105–108^. The probable processes through which dietary supplements support cardiovascular health are shown in Fig. 6.

Omega-3 fatty acids are a type of polyunsaturated fatty acid that is found in fatty fish, nuts, and seeds^109^. They have been shown to lower blood pressure and reduce the risk of heart disease^110^. The molecular mechanism by which omega-3 fatty acids regulate blood pressure is not fully understood, but it is thought to involve the inhibition of the production of inflammatory cytokines, which can cause the constriction of blood vessels and increase blood pressure^106^. Omega-3 fatty acids have also been shown to reduce levels of triglycerides and LDL cholesterol and increase levels of HDL cholesterol^110^ which can help to prevent the buildup of plaque in the arteries and reduce the risk of heart disease^111^.

Fruits, vegetables, and tea all include a group of substances known as flavonoids^112^. Research by Ohishi et al*.*^113^ has demonstrated that they contain anti-inflammatory and antioxidant characteristics as well as the ability to lower the risk of cardiovascular disease. The activation of the endothelial nitric oxide synthase enzyme, which can relax blood arteries and lower blood pressure, is assumed to be the molecular mechanism by which flavonoids modulate blood pressure^114^. Furthermore, flavonoids have been shown to increase HDL cholesterol levels and decrease triglyceride and LDL cholesterol levels^107^.

In fruits, vegetables, and tea, a group of substances known as polyphenols can be discovered^115^. They have been shown to have antioxidant and anti-inflammatory properties, and to reduce the risk of cardiovascular disease^116^. The molecular mechanism by which polyphenols regulate blood pressure is thought to involve the inhibition of the renin–angiotensin–aldosterone system, which can cause the constriction of blood vessels and increase blood pressure^114^. Similar to flavonoids, polyphenols have also been shown to reduce levels of LDL cholesterol and triglycerides and to increase levels of HDL cholesterol^107^.

Therefore, nutraceuticals such as omega-3 fatty acids, flavonoids, and polyphenols have been found to play a role in promoting cardiovascular health by regulating blood pressure, cholesterol levels, and inflammation. The molecular mechanisms by which these compounds exert their beneficial effects are not fully understood, but they are thought to involve the inhibition of inflammatory cytokines, the activation of endothelial nitric oxide synthase, and the inhibition of the renin–angiotensin–aldosterone system.

#### Nutraceuticals in neurodegenerative diseases

**Figure 7:**
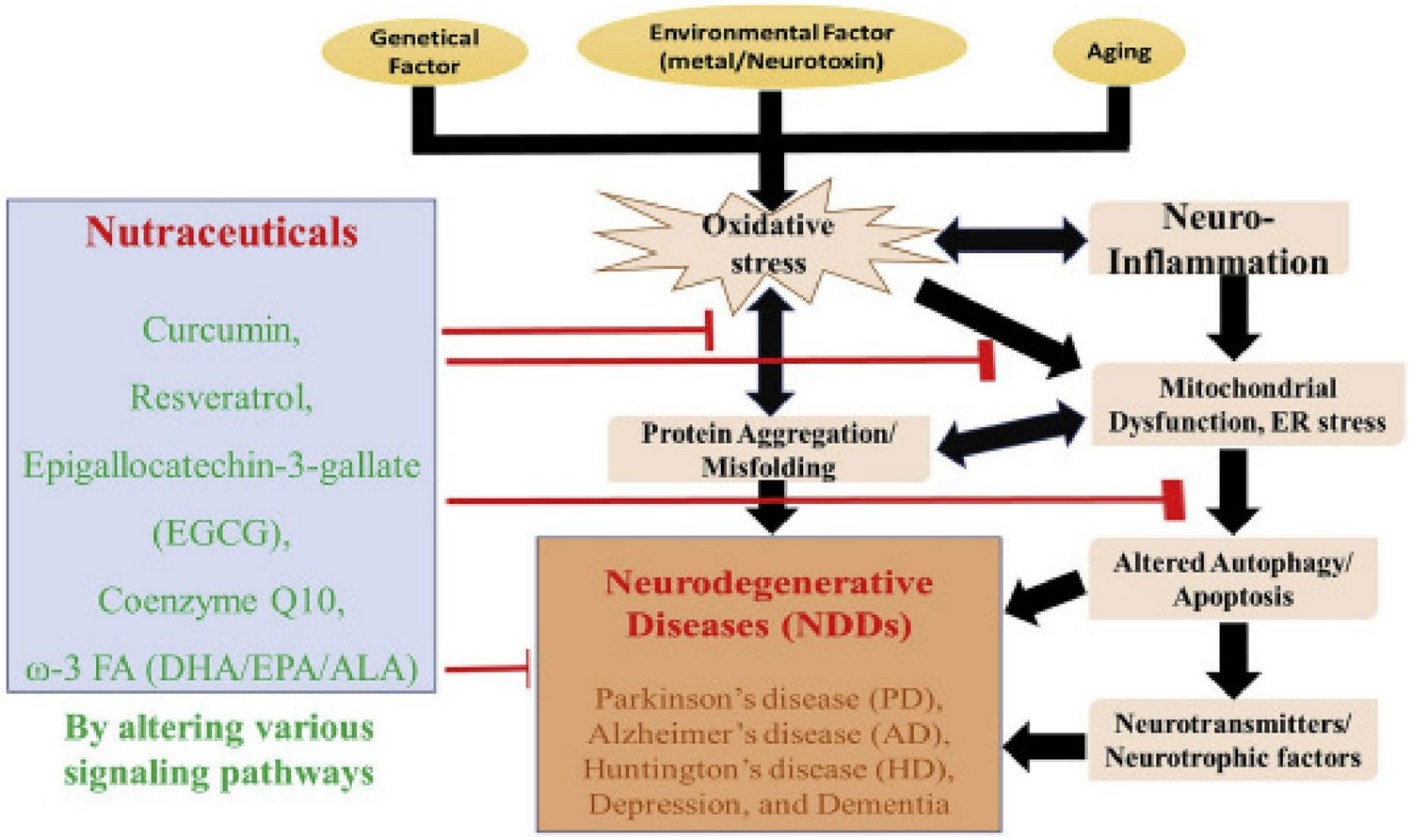
Molecular mechanisms of nutraceuticals in neurodegenerative diseases^117^.

Nutraceuticals have been studied for their potential role in preventing and treating neurodegenerative diseases such as Alzheimer's, Parkinson's, etc.^117–119^ (Fig. 7).

According to Chiu et al*.*^117^, oxidative stress is one of the molecular targets for nutraceuticals in neurodegenerative disorders. This happens with the emergence of an imbalance between the body’s ability to use antioxidants to neutralize reactive oxygen species (ROS) and their synthesis^119,120^. According to Teter et al*.*^121^, ROS has been connected to the emergence of neurodegenerative illnesses and can harm cellular components including proteins, lipids, and DNA.

Fish oil and flaxseed oil contain omega-3 fatty acids, which have been demonstrated to have anti-inflammatory and antioxidant characteristics, which can both aids prevent neurodegeneration^71^. According to studies^117,121^, omega-3 fatty acids may also enhance cognitive performance in persons with moderate cognitive impairment and lower the chance of acquiring Alzheimer’s disease.

Specifically, against Parkinson’s disease, flavonoids have been investigated for their possible neuroprotective properties^120^. Flavonoids have been demonstrated to affect signaling pathways involved in cell survival and death, as well as to have antioxidant and anti-inflammatory activities^117^. In animal models of Parkinson's disease, the flavonoid quercetin in particular has been demonstrated to shield neurons from harm and degeneration^118,120^.

Typically, oxidative stress, inflammation, and cell survival pathways are the molecular targets of nutraceuticals in neurodegenerative disorders. Nutraceuticals like flavonoids and omega-3 fatty acids have both been investigated for their possible neuroprotective properties^71,107^. They could influence these targets by lowering inflammation and oxidative stress while enhancing cell survival pathways^117^.

#### Nutraceuticals and gut health

Numerous areas of health, including gut health, can be supported by nutritional supplements^122^. The collection of microbes that live in the human gastrointestinal system, or the gut microbiome, is essential to gut health^123,124^. Nutraceuticals including probiotics, prebiotics, and fiber can all help control the gut microbiota and immune system^106^.

**Figure 8:**
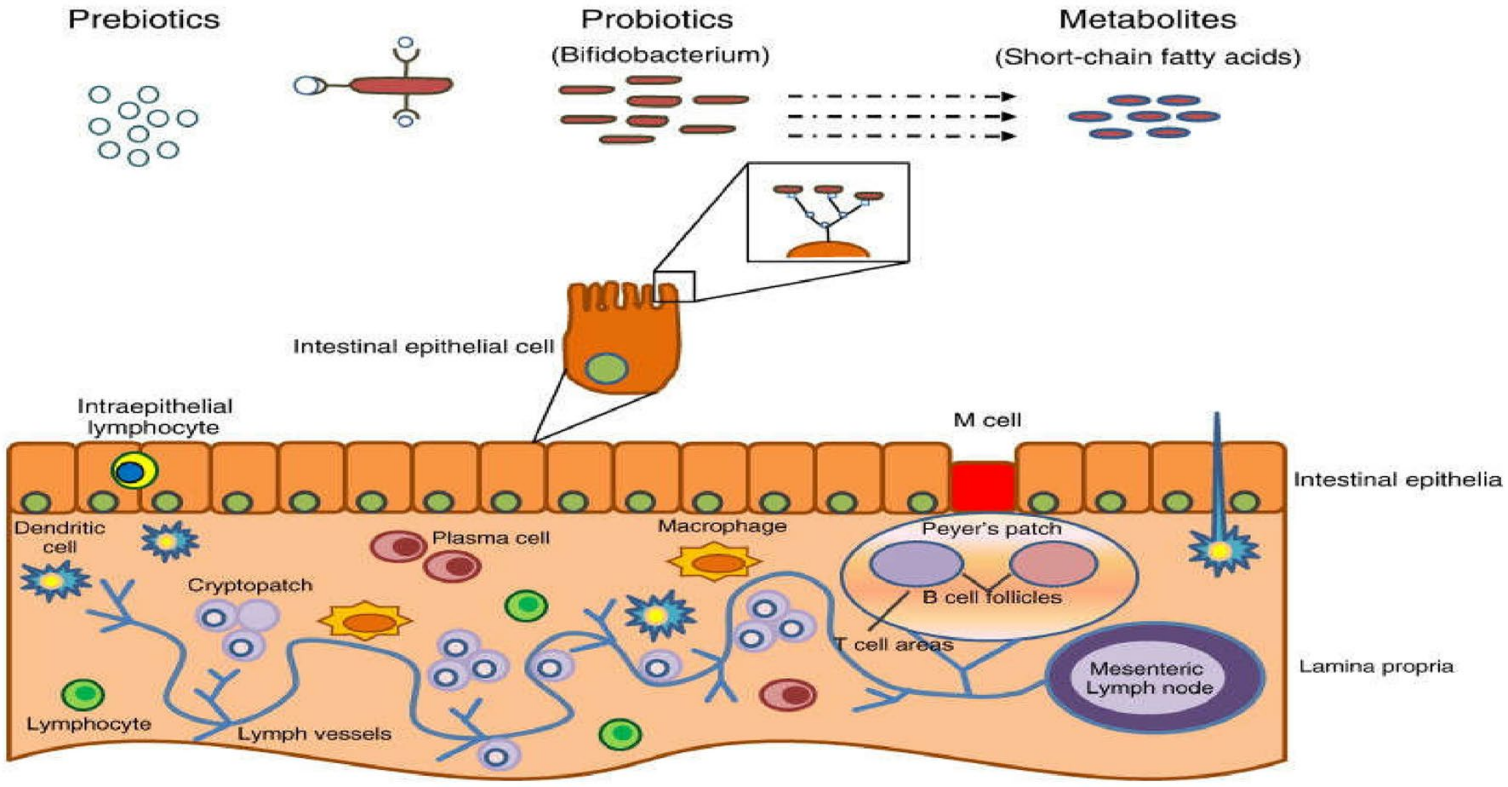
Mechanism of prebiotic actions^125^.

Figure 8 illustrates how probiotics, which are living microbes, can help the host's health when given in sufficient doses. According to Abdulhussein et al*.*^125^, probiotics can alter the makeup and operation of the gut microbiome and enhance gut health. For instance, it has been demonstrated that certain probiotic strains can boost immunological function, increase gut barrier function, and reduce gut inflammation^126^.

Probiotics have many different and intricate molecular processes that contribute to their positive benefits^106^. Through some signaling pathways, including toll-like receptors (TLRs), nucleotide-binding oligomerization domain (NOD)-like receptors, and pattern recognition receptors (PRRs), probiotics can interact with immune cells and gut epithelial cells^126^. Additionally, probiotics can generate many metabolites that influence the immune system and gut flora (Fig. 8).

Prebiotics are non-digestible dietary components that support the development and activity of healthy bacteria in the gut. By boosting the number of advantageous bacteria in the gut microbiome, such as Bifidobacteria and Lactobacilli, prebiotics can enhance gut health^126^. Short-chain fatty acids (SCFAs), which have anti-inflammatory characteristics and can enhance gut barrier function, can also be produced by prebiotics^106^. Prebiotics’ interaction with the gut microbiota is one of the molecular processes behind the positive benefits of prebiotics^127^. Prebiotics can specifically encourage the development of good bacteria that can create SCFAs, which can alter the gut microbiota and immune response^122^.

According to Das et al*.*^128^, fiber is a kind of complex carbohydrate that is not broken down by human enzymes and can serve as a prebiotic. For instance, fiber can enhance gut health by boosting the generation of SCFAs, accelerating gut motility, and encouraging the proliferation of advantageous bacteria in the gut microbiome^106,126^. Additionally, poisons and other hazardous chemicals in the gut can be bound by fiber and eliminated from the body. The chemical processes that underlie fiber's beneficial effects are equally intricate and varied. Prebiotics that resemble fiber can specifically encourage the growth of good bacteria that can create SCFAs, which can alter the gut microbiota and immune response. The release of many gut hormones, including peptide YY (PYY) and glucagon-like peptide 1 (GLP-1), which control gut motility and hunger, can also be stimulated by fiber^128,129^. Furthermore, according to Das et al*.*^128^, fiber can bind to bile acids and other toxic chemicals in the gut and remove them from the body.

Probiotics and prebiotics, like fiber, are nutraceuticals that can promote gut health and help control gut flora. The intricate molecular processes behind their beneficial effects include many signaling channels and metabolites. People can boost their gut health and general wellbeing by including these nutraceuticals in a balanced diet.

#### Nutraceuticals and sickle cell disorder

**Figure 9:**
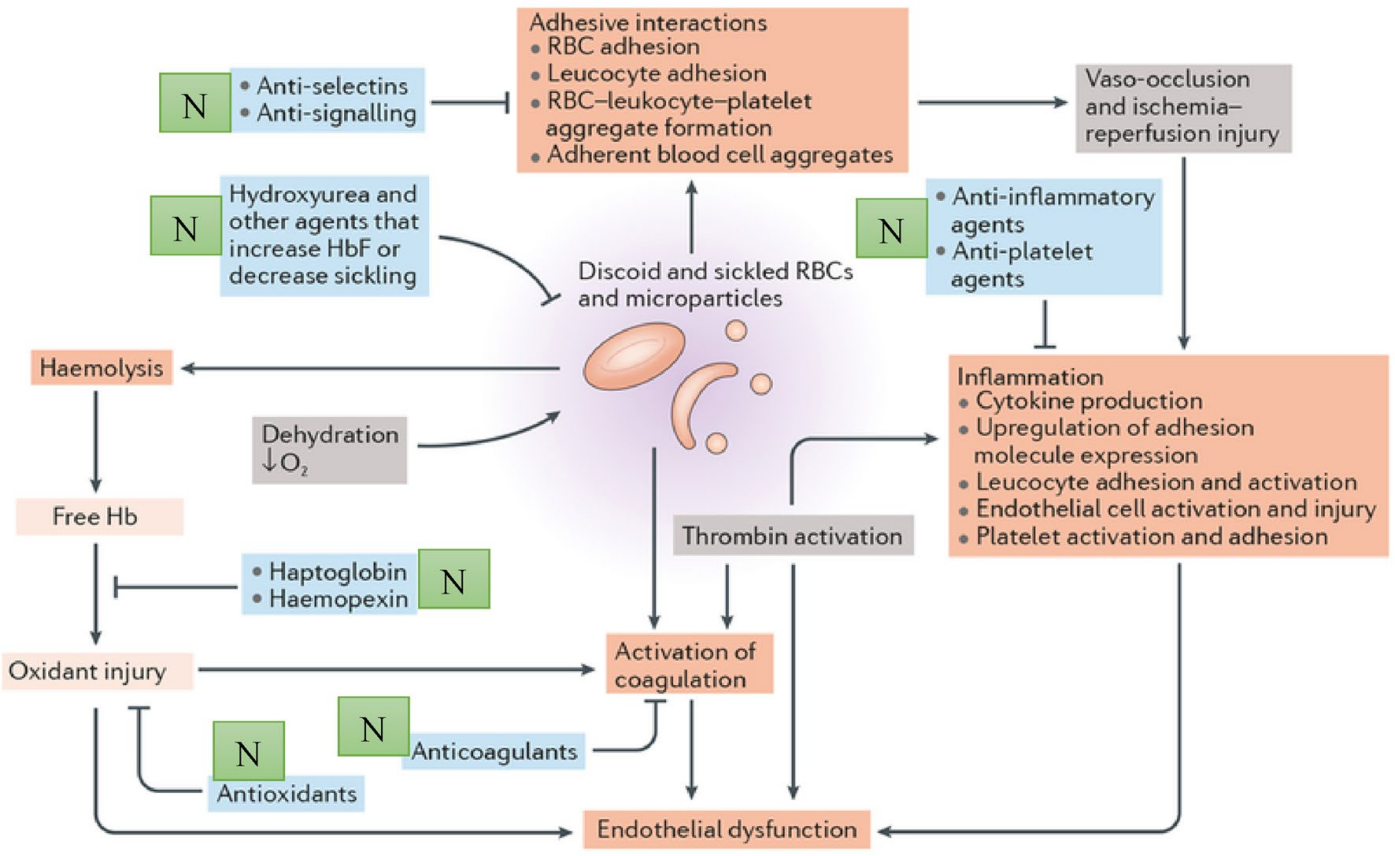
Mechanisms of nutraceuticals in sickle cell diseases^135^ with modifications (N = Nutraceuticals: Curcumin, Resveratrol, Quercetin, N-Acetyl cysteine, etc.).

Red blood cells with an irregular shape are thought to contribute to sickle cell disease (SCD), which can result in discomfort and organ damage due to blockages in tiny blood arteries^130,131^. Due to their alleged anti-inflammatory, antioxidant, and antiplatelet effects, nutraceuticals have been researched as a possible anti-sickling chemotherapeutic drug in SCD^132–134^. The molecular targets of nutritional supplements in sickle cell disorders are depicted in Fig. 9.

Resveratrol, a phytonutrient that is present in grapes and other fruits, is one nutraceutical that has been investigated for SCD^134^. Resveratrol has been demonstrated to lessen oxidative stress, inflammation, and platelet activation, all of which are implicated in the pathophysiology of SCD^135^. Resveratrol oral administration decreased inflammatory and oxidative stress markers in a study of SCD patients, pointing to a potential preventive benefit against SCD consequences^136,137^.

A flavonoid present in many fruits and vegetables called quercetin is another nutraceutical that has demonstrated potential as an anti-sickling agent^138^. Quercetin has been proven to increase red blood cell deformability and decrease sickling in red blood cells when tested in vitro^139,140^. These effects may assist avoid blockages in tiny blood arteries. Al Balushi et al*.*^132^ observed that quercetin also has anti-inflammatory and antioxidant properties that may aid to lessen the consequences of SCD problems.

A component of turmeric called curcumin has also been investigated for its possible anti-sickling properties^137^. The pathophysiology of SCD is influenced by oxidative stress, inflammation, and platelet activation, all of which can be reduced by curcumin (Vona et al*.*^136^). Curcumin administration reduced inflammatory and oxidative stress indicators in a study of SCD patients and improved blood flow, suggesting a possible preventive benefit against SCD consequences^137,141^.

Nutraceuticals have therefore demonstrated potential as anti-sickling chemotherapeutic agents in SCD because of their alleged anti-inflammatory, antioxidant, and antiplatelet characteristics^142^. Several dietary supplements have been investigated for their potential to offer protection against the side effects of SCD, including resveratrol, quercetin, and curcumin. The best dosages, formulations, and supplementation duration for these substances, as well as their effectiveness and safety in bigger clinical studies, need to be determined via more studies.

#### Nutraceuticals in reproductive health

**Figure 10:**
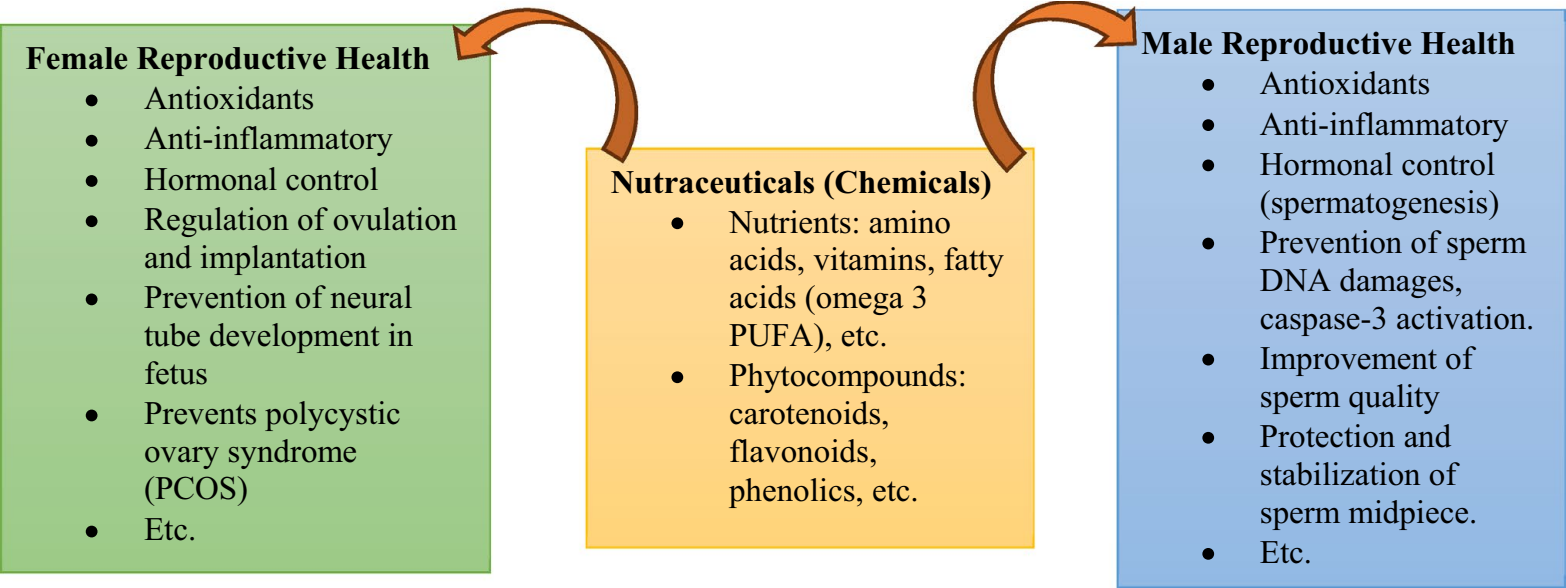
Actions of the Nutraceuticals on reproductive health.

The use of nutraceuticals as possible chemotherapeutic agents to enhance both male and female reproductive health has grown in prominence in recent years^143^ (see Fig. 10). According to research by Ma et al*.*^144^, these items include natural substances that have been demonstrated to enhance sexual function, hormonal balance, and fertility. Examples of these substances include vitamins, minerals, amino acids, and phytochemicals.

Nutraceuticals contained in natural goods like seed oil formulations, such as L-carnitine, CoQ10, zinc, and phytocompounds, have been demonstrated to increase sperm count, motility, and morphology for male reproductive health^78,144^. These substances have antioxidant properties that shield sperm from oxidative stress and enhance mitochondrial performance^145^. Men’s sexual drive and performance have also been demonstrated to be enhanced by nutraceuticals containing maca root extract^146^.

Nutraceuticals including folic acid, vitamin D, and omega-3 fatty acids have been demonstrated to enhance female fertility and hormonal balance. Vitamin D is important for ovulation and implantation whereas folic acid helps to prevent neural tube abnormalities in growing babies^147^. Omega-3 fatty acids may also boost fertility by regulating hormone levels and lowering inflammation in the reproductive system^148^.

In general, the use of nutraceuticals as possible chemotherapeutic agents has demonstrated positive outcomes for enhancing both male and female reproductive health^78,147^. A starting point for the identification of innovative medication formulations is the invalidation of these chemotherapeutic actions of nutraceuticals through the use of molecular docking. While research continues to fully understand the usefulness and safety of nutraceuticals, using these natural substances in a balanced diet may aid those who struggle with infertility or hormone imbalances^148^. However, it's crucial to speak with a doctor before including nutraceuticals in your diet, especially if you take any drugs or have underlying medical issues.

## Conclusion

Molecular docking is a useful approach for identifying the molecular targets of nutraceuticals in the treatment of illness. To identify possible treatment targets, it enables the prediction of the binding affinity and conformation of nutraceuticals with target proteins. The availability of databases and the improvement of computational tools have made molecular docking a crucial tool in the drug discovery process. The usage of this technology has improved drug discovery's efficiency and efficacy by cutting the time and expense needed for conventional experimental procedures. Therefore, the use of molecular docking in research on dietary supplements has considerable potential for the identification of novel therapeutic targets and the creation of secure and efficient dietary supplements for the treatment of disease.

## Future perspectives

A potent approach for identifying the molecular targets of nutraceuticals in the treatment of illness is molecular docking. Future developments in molecular docking tools and algorithms will improve the precision and effectiveness of discovering prospective nutraceutical targets. Additionally, molecular docking will be combined with other computational techniques like network analysis and machine learning to provide a more thorough knowledge of the complicated interactions between nutraceuticals and their molecular targets. Additionally, the use of molecular docking in personalized medicine will enable the development of customized treatment plans based on a person's genetic make-up and illness condition.

## Data Availability

Data relating to this review are available on request. Kindly contact the corresponding author to request the data from this study.
